# Table Tennis as a Sustainable Health Intervention: A Meta-Analysis of Its Effects on Balance and Cognitive Functions

**DOI:** 10.3390/healthcare14050675

**Published:** 2026-03-06

**Authors:** He Li, Hyunkyun Ahn, Minhye Shin

**Affiliations:** 1 Department of Physical Education, Sookmyung Women’s University, Seoul 04310, Republic of Korea; lihe02@sookmyung.ac.kr; 2 Department of Sport & Leisure Studies, Division of Arts & Health Care, Myongji College, Seoul 03656, Republic of Korea; 3 Department of Fitness Industry, Kyungil University, Gyeongsan 38428, Republic of Korea

**Keywords:** table tennis intervention, balance function, cognitive function, meta-analysis, sustainable health

## Abstract

**Background:** Physical inactivity is linked to falls, cognitive decline, and reduced independence, underscoring the need for accessible interventions that enhance balance and cognitive functions. In this context, sustainability refers to affordability, accessibility, feasibility, and potential for long-term adherence. Table tennis, an open-skill sport requiring motor coordination and cognitive engagement, may help improve balance and cognitive functions. This meta-analysis synthesized available evidence to investigate the effects of table tennis interventions on balance and cognitive functions. **Methods:** Following Preferred Reporting Items for Systematic Reviews and Meta-Analyses 2020 guidelines and PROSPERO registration, six electronic databases were searched. A meta-analysis was conducted with 14 randomized controlled trials comprising 1565 participants in total. Additionally, subgroup analyses were conducted based on intervention participants, health status, intervention duration, and session length. **Results:** Pooled analyses showed that individuals who received table tennis interventions demonstrated significantly improved balance (standardized mean difference [SMD] = 0.78, 95% confidence interval [CI] [0.57, 0.98]) and cognitive (SMD = 2.05, 95% CI [1.27, 2.83]) functions than those who did not. Subgroup analyses indicated consistent benefits across different age groups and health statuses, along with larger cognitive function effects with longer interventions. Despite considerable heterogeneity across studies and limited evidence for some subgroups, sensitivity analyses supported the robustness of the results. **Conclusions:** Table tennis appears to be a feasible and low-cost intervention that can effectively enhance balance and cognition functions, with potential applicability in community, educational, and rehabilitation settings. However, considering the observed heterogeneity and methodological limitations, the findings should be interpreted with caution.

## 1. Introduction

Insufficient physical activity (PA) is one of the most pressing public health concerns in the 21st century [1,2]. According to the World Health Organization (WHO), physical inactivity causes > 3.2 million deaths each year worldwide, making it the fourth most significant risk factor for global mortality [3,4]. Additionally, it is closely associated with cardiovascular diseases, increased risk of falls, cognitive decline, dementia, and mental health issues [5,6,7,8]. Balance and cognitive functions are critical determinants of independent living, risk of falls, and cognitive deterioration. Compared with other physical fitness components such as muscular strength or cardiorespiratory endurance, impairments in balance and cognitive functions are more directly linked to functional independence, fall-related injuries, and progression to disability, particularly in aging populations and individuals with health conditions. Therefore, balance and cognitive function are clinically meaningful and functionally relevant indicators of health, substantiated in previous research [9,10,11].

Given the rapid population aging worldwide, the WHO has emphasized the need for affordable, feasible, and sustainable PA interventions that enhance balance and cognitive functions, thereby creating a healthier society [12,13]. Regular PA can enhance both balance and cognitive functions [14,15,16,17,18]. Interventions involving dance, Tai Chi, and aerobic exercise have shown beneficial effects [19,20,21,22,23]. However, their effectiveness has varied across studies and dependent variables, partly due to differences in intervention dosage (e.g., frequency and duration), population characteristics (e.g., age and health status), and outcome measurement methods. For instance, dance interventions improve balance capacity; however, results regarding their effects on the reaction-time component of cognitive function are mixed [24]. Furthermore, many interventions have focused on a single outcome. For example, tennis interventions have mainly targeted cognitive function [25,26], whereas balance functions have received comparatively limited attention. This discrepancy may reflect the use of standardized neuropsychological assessments for cognition, which facilitate cross-study comparisons, while balance functions are more often examined in interventions explicitly designed to challenge postural control. Nevertheless, balance and cognitive functions are closely interconnected through shared neural mechanisms. Postural control is not entirely automatic but relies on attentional and executive resources. Dual-task research demonstrates that concurrent cognitive and balance tasks often interfere with one another, reflecting shared processing demands. In addition, balance requires continuous integration of multisensory inputs, which involves cognitive regulation. These findings indicate that balance and cognition are closely linked, supporting the need for their simultaneous evaluation. Therefore, identifying exercise modalities capable of simultaneously improving both domains is necessary.

Given the variability and domain-specific focus observed in existing interventions, the simultaneous influence of certain exercise modalities on both balance and cognitive functions should be clarified. Table tennis is one such promising modality. It is a multimodal activity that engages motor and cognitive processes simultaneously. Because these demands occur in parallel during play, improvements may emerge in both domains rather than in isolation. As a sport that requires rapid coordination, agility, dynamic balance, quick reaction time [27], tactical decision-making, spatial perception, and attentional shifting, it imposes physical and cognitive demands on participants. Table tennis interventions can enhance attention, visual perception, response inhibition, and executive function [28,29], while improving motor outcomes such as single-leg stance, postural stability, and motor coordination [27,30]. Moreover, table tennis is widely regarded as an affordable and accessible sport suitable for participants of all ages. With over 350 million players globally, it is among the most widely practiced sports worldwide [31]. Its low equipment costs and minimal space requirements make it feasible for implementation in community centers, schools, and rehabilitation settings [31]. These findings suggest that table tennis interventions may help reduce the long-term healthcare burden associated with falls and cognitive decline. Additionally, they have been regarded as sustainable interventions that are consistent with the United Nations Sustainable Development Goal 3, which aims to ensure healthy living and promote well-being for all at all ages [32].

Although individual studies have reported beneficial effects, the overall magnitude, robustness, and consistency of these effects remain unclear. Differences in sample sizes, study designs, intervention characteristics, and outcome measures make it difficult to draw definitive conclusions regarding the effectiveness of table tennis interventions. Existing meta-analyses have primarily focused on their effectiveness in improving vision or cognition [33,34], while systematic reviews have often targeted child populations [28]. To date, no meta-analysis has simultaneously examined the effects of table tennis interventions on both balance and cognitive functions. A quantitative synthesis that integrates findings across studies is necessary to estimate pooled effect sizes, evaluate overall patterns, and verify consistent benefits across both domains. This dual focus on balance and cognition is crucial, as these two functions are closely linked and jointly influence independence and fall risk, particularly in older adults and individuals with specific health conditions [31,35]. Evaluating the effects of table tennis interventions across both functions can provide more comprehensive evidence to support health interventions and further clarify the potential role of table tennis as a health-promoting activity.

To address this gap, we conducted a meta-analysis to quantitatively synthesize existing empirical evidence on the effects of table tennis interventions on balance and cognitive function. The primary outcomes were changes in balance and cognitive function, while secondary analyses explored whether the intervention effects varied according to intervention characteristics (intervention duration and session length), target population demographics, and health status. Through this approach, we aimed to evaluate table tennis as a sustainable health intervention and provide comprehensive evidence for health promotion.

## 2. Methods

This review was conducted in accordance with the 2020 Preferred Reporting Items for Systematic Reviews and Meta-Analyses (PRISMA) guidelines [36] and was prospectively registered in the PROSPERO database (CRD420251114980; registered on 4 August 2025).

### 2.1. Search Strategy

We developed a systematic search strategy to identify studies examining the effects of table tennis interventions on balance and cognitive functions. We searched for such studies on the Web of Science Core Collection, Embase, PubMed, Research Information Sharing Service (RISS), China National Knowledge Infrastructure (CNKI), and WanFang databases, covering all records from database inception to July 2025. No publication date restrictions were applied. The final search was completed on 31 July 2025. The following search terms were used: ((pingpang OR pingpong OR “table tennis”) AND ((cognitive OR cognition OR “cognitive function” OR “cognitive performance” OR memory OR “executive function” OR “reaction time”) OR (balance OR “balance function” OR “dynamic balance” OR “static balance” OR “postural balance”))). In PubMed, free-text terms were combined with MeSH headings to enhance sensitivity, and the search syntax was adapted for other databases. The reference lists of relevant reviews were manually screened to minimize potential omissions.

### 2.2. Inclusion and Exclusion Criteria

The selection of eligible studies was guided by the Population, Intervention, Comparison, Outcome, and Study design (PICOS) framework. Regarding the target population (P), we included studies focusing on children, adults, or older adults, including both healthy and non-healthy populations. Regarding the intervention (I), we included studies involving conventional, in-person, coach-led table tennis training, excluding virtual or exergame-based formats. Regarding the comparison group (C), we incorporated studies with a control group that did not engage in table tennis training. Regarding the outcome (O), we included studies in which at least one outcome measure was directly related to balance or cognitive functions. In this meta-analysis, balance function was conceptualized as a multidimensional motor ability reflecting the capacity to maintain postural stability under static or dynamic conditions. Accordingly, studies employing validated measures of static balance, dynamic balance, postural control, or functional balance performance were eligible for inclusion. Cognitive function was conceptualized as a broad, multidimensional construct encompassing multiple cognitive domains. Therefore, studies using validated assessments of executive function, attention, working memory, reaction time, global cognitive performance, or cognitively demanding performance-related tasks were included. This inclusive approach reflects the multifaceted nature of cognitive function and aligns with previous exercise-based meta-analyses synthesizing heterogeneous cognitive outcomes. Regarding the study design (S), we incorporated randomized controlled trials (RCTs) reporting complete pre- and post-intervention data. We excluded conference abstracts, dissertations, theses, review articles, non-RCTs, studies with incomplete or non-extractable data, studies in which the intervention involved table tennis video games or virtual reality table tennis, and studies not written in Korean, Chinese, or English.

### 2.3. Study Selection and Data Extraction

Two reviewers independently screened studies according to the inclusion and exclusion criteria. First, the titles and abstracts were screened to exclude irrelevant studies. Next, the reviewers assessed the full text of potentially eligible articles. The authors independently extracted data from the included studies in accordance with quality standards. In particular, publication year, author details, participant characteristics, intervention details, outcome measures, and study results were extracted from the selected studies. In this process, although formal inter-rater reliability statistics (e.g., Cohen’s kappa) were not calculated, disagreements were resolved through discussion with a third reviewer to ensure consensus.

### 2.4. Heterogeneity, Sensitivity Analysis, and Risk of Bias Assessment

Based on Cochrane methodology, heterogeneity was assessed using the I^2^ statistic. An I^2^ value of <25% indicated low heterogeneity, 25–50% indicated moderate heterogeneity, and >50% indicated high heterogeneity [37]. For continuous outcomes, a random-effects model was applied when I^2^ exceeded 50% [38].

A leave-one-out sensitivity analysis was conducted to evaluate the robustness of the pooled effect estimates [39]. One study was removed at a time, and the meta-analysis was re-performed to determine whether any single study exerted a disproportionate influence on the overall results.

The Cochrane Risk of Bias Tool was employed to assess potential bias [40], which evaluates RCTs based on six domains: random sequence generation, allocation concealment, blinding of participants and personnel, blinding of outcome assessment, incomplete outcome data, and selective reporting. For each domain, the RCT was classified as having low, unclear, or high risk of bias. Considering that blinding of participants is often impractical in sports-related RCTs [41], only studies explicitly reporting single- or double-blinding were rated as low risk; those not reporting blinding were classified as unclear risk. This classification was based on the Cochrane Risk of Bias guidelines, which recommend assigning an “unclear” rating when insufficient information is provided to permit a definitive judgment, rather than assuming a “high” risk in the absence of explicit reporting. If a study exhibited serious bias in any domain, it was assigned an overall “high” risk, whereas an overall “low” risk of bias required all domains to be rated as low risk [42]. The risk of bias was evaluated independently by two reviewers, and disagreements were resolved by involving a third reviewer.

### 2.5. Data Analysis

The data were analyzed using Review Manager version 5.3 (Cochrane, London, UK). During data extraction, special attention was given to the directionality of the outcome measures. Some outcomes, such as the Hasegawa’s Dementia Scale score for cognitive function, were coded such that higher values indicated better performance, whereas others, such as Total Errors, were coded such that lower values indicated better performance. To ensure consistency in the direction of effect sizes across studies, all outcomes were transformed prior to analysis so that positive values consistently indicated a beneficial effect of the intervention. We multiplied the pre- to post-intervention mean difference (MD) by −1 for outcomes in which lower scores indicated better performance [43].

Standardized mean differences (SMDs, Hedges’ g) were automatically calculated by RevMan based on pre- and post-intervention MDs and their standard deviations (SDs) [44]. The SD of the MD (SD_change) was calculated as follows [42].

SD_change = √(SD^2^_pre + SD^2^_post − 2 × r × SD_pre × SD_post)

In this equation, SD_change represents the SD of the mean change, SD_pre and SD_post denote SDs at baseline and post-intervention, respectively, and r signifies the correlation coefficient between pre- and post-intervention measurements. As original studies have rarely reported this correlation, we adopted a fixed r value of 0.7 based on previous research [45].

Given the variations in measurement methods for balance and cognitive function across the included studies, we computed SMDs with 95% confidence intervals (CIs) and applied a random-effects model to evaluate the effects of table tennis interventions. The use of SMDs allowed the integration of outcomes derived from different balance and cognitive domains and assessment tools, while the random-effects model accounted for both methodological and conceptual heterogeneity across studies. According to Cohen’s criteria, SMDs of approximately 0.2, 0.5, and 0.8 indicate a small, moderate, and large effect size, respectively [46].

Subgroup analyses were pre-specified by population (children vs. older adults), health status (healthy vs. non-healthy), intervention duration (≤12 vs. >12 weeks), and session length (≤60 vs. >60 min), as these factors may affect responses to table tennis training.

## 3. Results

### 3.1. Study Selection

**Figure 1:**
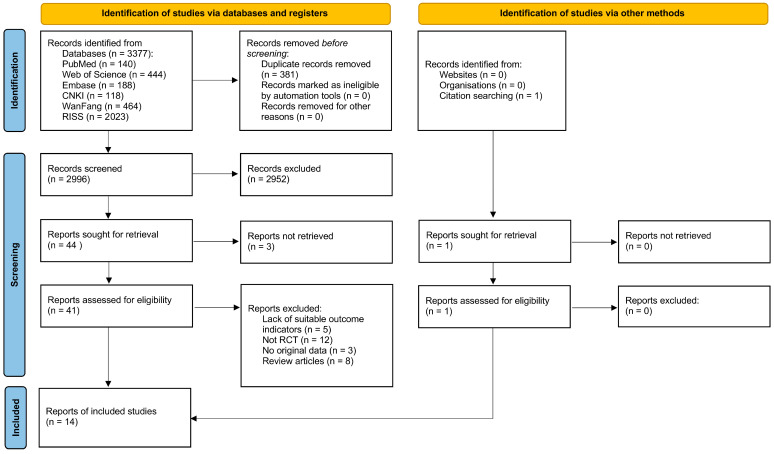
Flow diagram of study selection.

A total of 3378 candidate studies were retrieved through the literature search. After removing 381 duplicates, the titles of 2997 records were screened. Of these, 2894 studies were excluded owing to irrelevance to the research topic. Then, the abstracts of 103 eligible studies were reviewed, resulting in the exclusion of 59 studies. The full texts of the remaining 44 studies were assessed for eligibility, and 30 were excluded because they were non-RCTs (*n* = 12) or review articles (*n* = 8), lacked appropriate outcome indicators (*n* = 5), had inaccessible full text (*n* = 3), or lacked original data (*n* = 2). Ultimately, 14 articles met the inclusion criteria and were incorporated in the pooled analysis [30,47,48,49,50,51,52,53,54,55,56,57,58,59]. The PRISMA flow diagram (Figure 1) provides an overview of the procedures used for study screening and selection.

### 3.2. Characteristics of the Included Studies

The 14 included studies involved a total of 1565 participants, with 779 in the intervention group and 786 in the control group. The target population included children and adults, and healthy and non-healthy individuals. The studies were conducted in various countries, including South Korea, China, Iran, and Taiwan. All table tennis interventions were implemented under the supervision of qualified table tennis coaches. The length of the intervention varied between 8 weeks and 3 years, indicating a substantial variation in exposure duration, which could be a potential major contributor to inter-study heterogeneity. Participants underwent training two to five times per week. Each session lasted between 40 and 90 min. During the intervention, the intervention group engaged in table tennis training, whereas the control group did not receive such training. Table 1 summarizes the characteristics of the included studies.

### 3.3. Methodological Quality of the Included Studies

**Figure 2:**
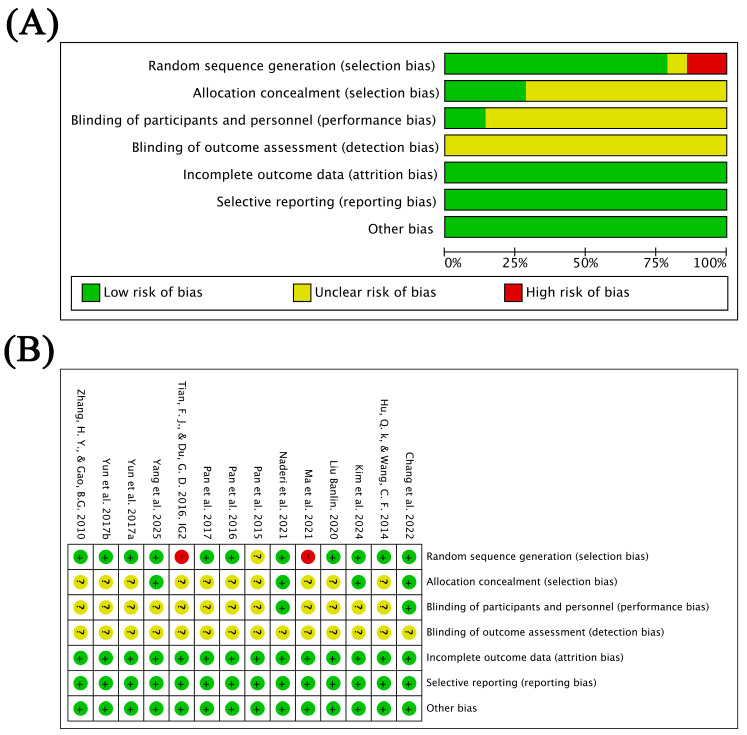
(**A**) Summarized risk of bias results; (**B**) Risk of bias results for each study, Green: low risk of bias; yellow: unclear risk of bias; red: high risk of bias, [30,47,48,49,50,51,52,53,54,55,56,57,58,59].

The quality of the 14 included studies was assessed using the Cochrane Risk of Bias tool. Regarding random sequence generation, 11 studies had low risk of bias, 1 had unclear risk of bias, and 2 had high risk of bias. Regarding allocation concealment, 4 studies had a low risk of bias and 10 had an unclear risk of bias. In terms of participant and staff blinding, 2 studies were judged to be at low risk of bias, whereas 12 were rated as unclear. None of the studies reported the blinding of outcome assessment. However, all studies had a low risk of bias concerning incomplete outcome data and selective reporting. Figure 2 presents the results concerning the risk of bias in the included studies.

### 3.4. Meta-Analysis for Balance Function

**Figure 3:**
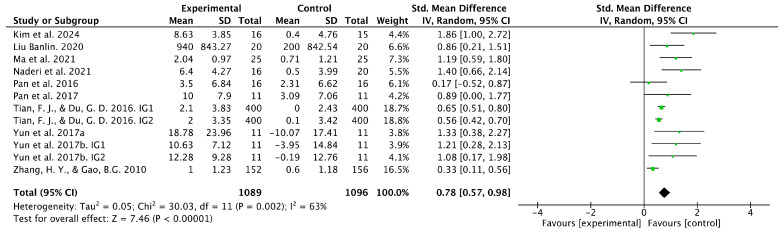
Forest plot showing the results of the meta-analysis for balance function. Green squares show the effect size of each study (square size reflects study weight), and the black diamond shows the pooled effect size with its 95% confidence interval. SD: standard deviation, CI: confidence interval, IG1: intervention group 1, IG2: intervention group 2, [30,48,49,50,52,53,54,56,57,58].

Ten studies investigated the effects of table tennis interventions on balance function. The pooled analysis showed a statistically significant difference in balance outcomes between the intervention and control groups (SMD = 0.78, 95% CI [0.57, 0.98], *p* < 0.001) (Figure 3). However, considerable heterogeneity was observed (I^2^ = 63%), indicating variability in effect sizes across studies. Therefore, a random-effects model was applied, followed by subgroup analyses to explore potential sources of heterogeneity. Sensitivity analysis, conducted by removing one study at a time, showed only minor changes in the pooled effect size, suggesting that no single study disproportionately influenced the overall estimate (Table 2). Nevertheless, considering the observed heterogeneity, the pooled effect size should be interpreted with caution.

### 3.5. Meta-Analysis for Cognitive Function

**Figure 4:**
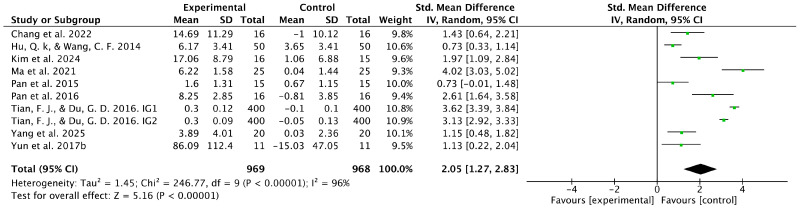
Forest plot showing the results of the meta-analysis for cognitive function. Green squares show the effect size of each study (square size reflects study weight), and the black diamond shows the pooled effect size with its 95% confidence interval. SD: standard deviation, CI: confidence interval, IG1: intervention group 1, IG2: intervention group 2, [47,49,50,51,53,55,56,58,59].

Nine studies examined the effects of table tennis interventions on cognitive function. The pooled analysis showed a statistically significant difference in cognitive outcomes between the intervention and control groups (SMD = 2.05, 95% CI [1.27, 2.83], *p* < 0.001) (Figure 4). However, substantial heterogeneity was observed (I^2^ = 96%), indicating marked variability in effect sizes across studies. Despite this high heterogeneity, a meta-analysis was considered appropriate because all included studies assessed conceptually related cognitive function outcomes following comparable table tennis interventions, and effect sizes were synthesized using SMDs to account for differences in measurement instruments. Therefore, a random-effects model was applied, followed by subgroup analyses to explore potential sources of heterogeneity. Sensitivity analysis, conducted by removing one study at a time, showed limited changes in the pooled effect size, suggesting that no single study disproportionately influenced the overall estimate (Table 3). Considering the magnitude of heterogeneity, the pooled estimate should be interpreted as reflecting an overall directional effect rather than a precise quantitative effect.

### 3.6. Subgroup Analyses

Table 4 presents the results of subgroup analyses based on intervention population, health status, intervention duration, and session length.

#### 3.6.1. Subgroup Analyses for Balance Function

In the subgroup analysis based on intervention population, the pooled effect size was SMD = 0.74 (95% CI [0.23, 1.26], *p* = 0.005) for children and SMD = 0.80 (95% CI [0.59, 1.01], *p* < 0.001) for older adults. These findings suggest a statistically significant association between table tennis interventions and improved balance function in both children and older adults.

In the subgroup analysis based on health status, the pooled effect size was SMD = 0.72 (95% CI [0.53, 0.92], *p* < 0.001) for healthy individuals and SMD = 0.95 (95% CI [−0.04, 1.94], *p* = 0.06) for non-healthy individuals. These results suggest that table tennis interventions significantly improved balance function in healthy individuals, whereas the effect among non-healthy individuals was not statistically significant according to conventional standards and should be interpreted cautiously owing to the limited number of studies.

Regarding intervention duration, the pooled effect size was SMD = 0.88 (95% CI [0.47, 1.30], *p* < 0.001) for interventions lasting ≤12 weeks and SMD = 0.73 (95% CI [0.51, 0.95], *p* < 0.001) for longer interventions. These findings suggest that improvements were observed across different intervention durations; however, inter-study variability should be considered while interpreting these subgroup estimates.

Regarding session length, sessions lasting ≤60 min yielded an SMD of 0.84 (95% CI [0.37, 1.31], *p* = 0.005), whereas longer sessions yielded an SMD of 1.06 (95% CI [0.32, 1.80], *p* = 0.005). These findings indicate that table tennis interventions significantly enhanced balance function regardless of session length; however, numerically larger effect sizes were observed for longer sessions, although subgroup comparisons should be interpreted cautiously (i.e., those exceeding 60 min).

#### 3.6.2. Subgroup Analyses for Cognitive Function

The subgroup analysis for cognitive function showed similar results as that for balance function. Regarding the intervention population, the pooled effect size was SMD = 1.64 (95% CI [0.87, 2.41], *p* < 0.001) for children and SMD = 2.30 (95% CI [1.31, 3.29], *p* < 0.001) for older adults. These findings indicate that table tennis interventions enhanced cognitive function in both age groups, with greater improvements among older adults.

Regarding health status, the pooled effect size was SMD = 2.65 (95% CI [1.89, 3.41], *p* < 0.001) for healthy individuals and SMD = 1.42 (95% CI [0.74, 2.10], *p* < 0.001) for non-healthy individuals. These results suggest that table tennis interventions significantly improved cognitive function in healthy and non-healthy individuals.

Regarding intervention duration, the pooled effect size was SMD = 1.32 (95% CI [0.84, 1.79], *p* < 0.001) for interventions lasting ≤12 weeks and SMD = 3.46 (95% CI [3.02, 3.91], *p* < 0.001) for longer interventions. These findings indicate that both short- and long-term interventions significantly improved cognitive function, with numerically larger effect sizes in long-term interventions; however, these estimates were derived from a limited number of studies and should be interpreted cautiously.

The subgroup analysis based on session length yielded an SMD of 0.96 (95% CI [0.66, 1.26], *p <* 0.001) for sessions lasting ≤60 min and an SMD of 1.74 (95% CI [0.62, 2.85], *p* = 0.002) for longer sessions. These findings demonstrate that table tennis interventions significantly enhanced cognitive function regardless of session length, but longer sessions (i.e., those lasting greater than 60 min) showed more pronounced effects.

## 4. Discussion

In this meta-analysis, data from previous studies were pooled to evaluate the effects of table tennis interventions on balance and cognitive functions. In line with our methodological framework, balance function was conceptualized as a multidimensional motor ability encompassing static and dynamic postural control, whereas cognitive function was considered as a broad construct with multiple domains, including executive function, attention, working memory, reaction time, and global cognitive performance. Given the diversity of assessment tools used across studies, the pooled estimates should be interpreted as reflecting overall trends across related domains rather than effects on a single, unified construct. The findings suggest that table tennis interventions are associated with moderate improvements in balance function (SMD = 0.78) and substantial improvements in cognitive function (SMD = 2.05), both statistically significant. However, these estimates should be interpreted with caution, considering the methodological limitations identified in the included trials. To our knowledge, no previous meta-analysis has comprehensively examined the influence of table tennis interventions on balance and cognitive functions. Although the benefits associated with physical and open-skill activities have been well-documented in previous systematic reviews and meta-analyses [28,60,61], quantitative evidence specific to table tennis, which is characterized by rapid visuomotor coordination, complex motor sequences, and continuous cognitive engagement, remains limited.

### 4.1. Effects of Table Tennis Interventions on Balance Function

The pooled results indicated that table tennis interventions are associated with significant improvements in balance function. This finding aligns with the results of recent RCTs and narrative reviews reporting similar benefits [62,63]. Prior to this analysis, quantitative evidence was limited. For example, a systematic review (without meta-analysis) suggested that table tennis practice may enhance coordination capacity [28], but definitive conclusions could not be drawn. From a physiological perspective, balance (or postural control) relies on the integration of visual, vestibular, and proprioceptive inputs in the central nervous system, which subsequently generates motor commands to maintain an upright posture [64,65]. Visual input provides information about the surrounding environment, helping individuals detect changes in posture and orientation relative to external references, such as objects or the horizon. The vestibular system, located in the inner ear, detects head movements and helps maintain balance functions by sensing changes in acceleration and gravitational forces, which is critical for stabilizing posture during dynamic activities. Proprioception, or the body’s sense of position, arises from sensory receptors in muscles, joints, and skin, providing feedback to the brain about the body’s position and movement, thus supporting fine-tuned postural adjustments. Collectively, these systems work synergistically to generate appropriate motor responses, particularly during activities such as table tennis that require rapid, multidirectional footwork and anticipatory postural adjustments [65]. Previous research has examined the contributions of the sensory systems to postural control, highlighting the interplay of visual, vestibular, and proprioceptive inputs in maintaining balance function, especially in older adults [63]. Through frequent multidirectional footwork, rapid lateral movements, and anticipatory postural adjustments, table tennis training may stimulate vestibular function and reinforce neuromuscular control. Biomechanical studies of table tennis have shown that stroke execution involves rapid lateral accelerations, coordinated trunk–lower limb activation, and frequent postural adjustments, all of which involve continuous engagement of vestibular and neuromuscular systems [66]. These movement characteristics may partially explain the improvements in dynamic balance observed in our analysis. Additionally, the high demand for visual tracking and rapid motor responses resembles mechanisms targeted by interventions such as visual perturbation or VR-enhanced balance function training, which support sensory integration—a key determinant of dynamic balance function [67]. Taken together, these mechanisms may provide a plausible explanatory framework for the improved balance observed in our meta-analysis; however, they remain hypothetical and further empirical validation is needed. It should also be noted that balance function outcomes were assessed using different instruments across studies, including static and dynamic balance tests. Although these measures reflect related aspects of postural control, such methodological variability may have contributed to statistical heterogeneity and should be considered when interpreting the pooled results.

### 4.2. Effects of Table Tennis Interventions on Cognitive Function

Our analysis further demonstrated that table tennis interventions are associated with significant improvements in cognitive function, which aligns with previously reported findings [34]. Cognitive performance fundamentally depends on coordinated neural activity, and exercise-induced improvements are widely attributed to neuroplastic adaptations, such as neurogenesis, synaptogenesis, angiogenesis, elevated levels of neurotrophins such as brain-derived neurotrophic factor (BDNF), and enhanced task-related brain activation, particularly in regions such as the precuneus [68,69,70]. As a prototypical open-skill sport, table tennis requires continuous integration of motor, visual, and attentional processes in response to unpredictable stimuli. Players must rapidly alternate between inhibiting and initiating motor actions, demonstrating proactive inhibitory control while efficiently processing sensorimotor information [71]. Such demands may enhance neural sensitivity and inter-system coordination, potentially contributing to measurable cognitive improvements.

Recent neuroimaging evidence, primarily derived from observational and athlete-based studies, provides additional support for these potential mechanisms. Table tennis players exhibit more stable neural oscillations and enhanced functional connectivity in visual and sensorimotor regions, reflecting network patterns that balance function stability and flexibility to facilitate efficient information processing [72]. Moreover, long-term training is associated with improved white matter integrity (FA, AD) and strengthened dynamic functional connectivity between the orbitofrontal cortex (BA11), temporal lobe (BA48), and multiple cortical and subcortical regions, including the frontal, parietal, cerebellar, thalamic, and occipital lobes [73]. More importantly, some of these structural markers are correlated with visual attention performance. Overall, these findings suggest that structural and functional neuroplastic adaptations could underlie the cognitive improvements observed in this study. However, the included studies employed heterogeneous cognitive function assessments targeting different domains and performance characteristics. Although the use of SMDs allowed quantitative synthesis across measures, such conceptual and methodological variability may have contributed to the large pooled effect size and observed heterogeneity. Therefore, the present findings should be interpreted as indicating a general positive influence of table tennis on cognitive-related outcomes rather than domain-specific effects.

### 4.3. Effects of Table Tennis Interventions Stratified by Intervention Participants, Health Status, Intervention Duration, and Intervention Length

Subgroup analyses based on the intervention population revealed beneficial effects of table tennis interventions regardless of age, with older adults showing marginally greater improvements than children in both balance (SMD = 0.80) and cognitive functions (SMD = 2.30). Notably, there is a lack of direct evidence demonstrating that table tennis interventions are more effective in older adults than in children. However, previous studies have demonstrated that table tennis interventions lead to significant improvements among older adult populations [33]. These improvements may be attributable to lower baseline function, retained neuroplastic capacity despite age-related decline, and increased responsiveness to interventions. In contrast, children and adolescents experience natural improvements in neural and motor systems [74,75], which may attenuate the magnitude of intervention effects.

Subgroup analyses based on health status showed that table tennis interventions improve balance function in both healthy and non-healthy individuals. A significant pooled effect size (SMD = 0.72, *p* < 0.001) was evident in healthy individuals, whereas a higher but marginally significant effect (SMD = 0.95, *p* = 0.06) was observed in non-healthy individuals. Therefore, these findings suggest that table tennis interventions may hold a greater potential for improvement in non-healthy populations. However, this result should be interpreted cautiously, as only three studies with small sample sizes were included, resulting in wide CIs and limited statistical power. Large-scale randomized trials are warranted to confirm these effects and explore underlying mechanisms in special populations.

Regarding the intervention duration, interventions lasting ≤12 weeks and those lasting >12 weeks were both associated with significantly improved balance function (SMD = 0.88 and SMD = 0.73, respectively; both *p* < 0.001), suggesting that table tennis interventions enhance balance function irrespective of duration. However, unlike balance function outcomes, cognitive function outcomes appeared more dependent on intervention duration. Interventions lasting ≤12 weeks yielded an SMD of 1.32, whereas longer interventions demonstrated substantially larger improvements (SMD = 3.46). This pattern aligns with the findings of Behrendt et al. [76]. They observed that both open- and closed-skill exercises acutely elevate serum and plasma levels of neuroplasticity-related biomarkers (BDNF, IGF-1, IL-6), but only open-skill exercises maintain elevated baseline BDNF after 12 weeks of training. However, none of the studies included in the present meta-analysis reported biomarker data. Therefore, these biomarker dynamics remain speculative in the context of our findings. They may help explain the pronounced effects of long-term table tennis interventions on cognitive function, but this interpretation should be made with caution. Future studies should include direct measurements of these biomarkers to verify the proposed mechanisms more rigorously.

Regarding session length, compared with interventions comprising shorter sessions, those comprising sessions exceeding an hour produce slightly greater improvements in both balance and cognition functions. This suggests that increasing the length of sessions may further potentiate the benefits of table tennis interventions. Taken together, the effects of table tennis interventions appear to be both time-dependent and cumulative. They require consistent practice over several weeks or longer, rather than short-term engagement, to achieve optimal outcomes. This observation is consistent with the general characteristics of physical exercise [77,78]. However, several subgroup comparisons were based on a limited number of studies, particularly in non-healthy populations, long-term interventions (>12 weeks), and sessions exceeding 60 min. Therefore, these subgroup findings should be interpreted as exploratory rather than definitive and should not be overgeneralized.

### 4.4. Limitations

The present findings should be interpreted carefully, considering some limitations. First, considerable heterogeneity was observed across studies, which may be attributed to the wide age range of participants, variations in health status, inconsistencies in intervention frequency and duration, and diversity of outcome measures. In particular, cognitive function was assessed using various domain-specific and performance-related instruments, reflecting its multidimensional nature. Although standardized effect sizes were used to facilitate synthesis, such variability may limit the precision of domain-specific interpretations. Second, several subgroup analyses (e.g., non-healthy populations, long-term interventions, and longer session durations) were based on a limited number of studies, which may have resulted in unstable effect size estimates and an increased risk of overinterpretation. These subgroup findings should therefore be considered exploratory. Third, while examining intervention-related factors, this study considered only intervention duration and session length, without incorporating other key training parameters such as exercise intensity, weekly frequency, and progression strategies. These components are fundamental determinants of exercise dose and may substantially influence balance and cognitive adaptations. However, detailed reporting of training intensity (e.g., heart rate, percentage of maximal heart rate, or rating of perceived exertion), session frequency, and progression schemes was inconsistent or absent in many trials, precluding quantitative dose–response analyses. Future RCTs should adopt standardized reporting of these parameters to enable comparability across studies and facilitate more precise dose–response meta-analyses. Finally, although only RCTs were included, several studies provided insufficient details regarding allocation concealment and blinding procedures. Because blinding of participants and personnel is often difficult in exercise-based interventions, the possibility of performance bias cannot be excluded. In addition, inadequate reporting of allocation concealment may increase the risk of selection bias. These methodological limitations may have influenced the magnitude of the pooled effect estimates. Moreover, only studies published in English, Chinese, and Korean were included, which may have introduced language bias and limited the comprehensiveness of the evidence base. A few studies assessed both balance and cognitive outcomes with the same intervention; however, the small number and heterogeneity of these studies prevented a separate analysis of simultaneous effects. Future research should systematically evaluate both domains together.

## 5. Conclusions

This meta-analysis suggests that table tennis interventions can significantly improve both balance and cognitive functions across different populations. However, these findings should be interpreted with caution due to several methodological limitations, including considerable heterogeneity in study designs, small sample sizes, and limited reporting of allocation concealment and blinding procedures. These factors may introduce potential performance and selection bias and could have influenced the magnitude of the pooled effect estimates. Therefore, although statistically significant effects were observed, the overall strength of current evidence is constrained by methodological and reporting limitations.

Although table tennis shows promise as a low-cost and accessible open-skill activity, further rigorous evidence is needed to confirm its effectiveness and refine intervention protocols. Within this context, preliminary evidence suggests that table tennis may have practical relevance in various community settings. It could be a feasible option to support balance function in older adults, enhance attentional engagement in educational environments, and provide combined motor–cognitive stimulation in rehabilitation contexts. Nevertheless, these potential applications should be considered exploratory rather than prescriptive given the current limitations of the evidence base.

Future research should prioritize high-quality, large-sample RCTs with standardized reporting of training parameters and follow-up outcomes. Integrating physiological indicators and neuroimaging techniques may further elucidate the neuroplastic mechanisms underlying the observed benefits. Strengthening methodological consistency and reproducibility will be essential to establish robust conclusions regarding the clinical and public health implications of table tennis interventions.

## Figures and Tables

**Table 1:** Descriptive information of the included studies.

Year	Author	Number of Participants and Their Age and Gender	Intervention Program		Outcome Subtype	Results	*p*
2021	Naderi et al., [57]	N = 36 (CG = 20 and IG = 16) M = NA/F = NA >65 years	CG: No TT intervention	IG: Frequency: 3–5 times/week Type: TT exercises Session length: 90 min Intervention duration: 6 months	Balance function (Balance test in SPPB and Additional balance test)	Balance function differed significantly between the IG and CG	<0.001
2025	Yang et al., [51]	N = 40 (CG = 20 and IG = 20) M = NA/F = NA 65–70 years	CG:Health education but no TT intervention	IG: Frequency: 5 times/week Type: TT exercises Session length: 60 min Intervention duration: 12 weeks	Cognitive function (N-back working memory and Cognitive dual-task walking)	Cognitive function differed significantly	<0.005
2017a	Yun et al., [52]	N = 22 (CG = 11 and IG = 11) M = NA/F = NA 61–70 years	CG: Regular class but no TT intervention	IG: Frequency: 4 times/week Type: TT exercises Session length: 60 min Intervention duration: 12 weeks	Balance function (Static balance)	Balance function differed significantly between IG and CG	<0.01
2017b	Yun et al., [53]	N = 22 (CG = 11 and IG = 11) M = 8/F = 14 61–70 years	CG: Regular class but no PA intervention	IG: Frequency: 4 times/week Type: TT activities Session length: 60 min Intervention duration: 12 weeks	Cognitive function (Choice reaction time test), Balance function (Dynamic balance)	Cognitive function and dynamic balance function differed significantly between IG and CG	<0.05
2021	Ma et al., [49]	N = 50 (CG = 25 and IG = 25) M = 30/F = 20 ≥60 years	CG: Regular class but no PA intervention	IG: Frequency: NA Type: TT exercises Session length: NA Intervention duration: 6 months	Cognitive function (Hasegawa Dementia Scale), Balance function (TIME-SLC)	Cognitive function and balance function differed significantly between IG and CG	<0.05
2016	Tian and Du., [50]	N = 800 (CG = 400 and IG = 400) M = 497/F = 303 60–79 years	CG: Regular class but no PA intervention	IG: Frequency: 3 times/week Type: TT exercises Intervention duration: 3 years	Cognitive function (Choice reaction time), Balance function (TIME-SLC)	Cognitive function and balance function differed significantly between IG and CG	<0.05
2014	Hu and Wang., [47]	N = 100 (CG = 50 and IG = 50) M = 46/F = 54 56–78 years	CG: Regular class but no TT intervention	IG: Frequency: 3–4 times/week Type: TT exercises Session length: 60 min Intervention duration: 12 months	Cognitive function (Mini-Mental State Examination)	Cognitive function differed significantly be-tween IG and CG	<0.01
2022	Chang et al., [55]	N = 32 (CG = 16 and IG = 16) M = 26/F = 6 Children in Grade 1–6	CG: Regular class but no PA intervention	IG: Frequency: 3 times/week Type: TT exercises Session length: 60 min Intervention duration: 12 weeks	Cognitive function(Stroop Test)	Cognitive function differed significantly between IG and CG	<0.01
2016a	Pan et al., [58]	N = 32 (CG = 16 and IG = 16) M = NA/F = NA 6–12 years	CG: Regular class but no TT intervention	IG: Frequency: 2 times/week Type: TT exercises Session length: 70 min Intervention duration: 12 months	Cognitive function (Color-Word raw score), Balance function (Balance subtest standard score)	No significant difference in balance function but cognitive function differed significantly between IG and CG	<0.01
2016b	Pan et al., [30]	N = 22 (CG = 11 and IG = 11) M = 22/F = 0 6–12 years	CG: Regular class but no PA intervention	IG: Frequency: 2 times/week Type: TT exercises Session length: 70 min Intervention duration: 12 weeks	Balance function (BOT-2 Body Coordination Composite—Balance)	Balance function differed significantly between IG and CG	<0.01
2020	Liu, [48]	N = 40 (CG = 20 and IG = 20) M = NA/F = NA 6–9 years	CG: Regular class but no PA intervention	IG: Frequency: 2 times/week Type: TT exercises Session length: 60 min Intervention duration: 12 weeks	Balance function (Dynamic balance test)	Dynamic balance function differed significantly between IG and CG	<0.05
2015	Pan et al., [59]	N = 30 (CG = 15 and IG = 15) M = NA/F = NA 7–12 years	CG: Regular class but no PA intervention	IG: Frequency: 2 times/week Type: TT exercises Session length: 70 min Intervention duration: 12 weeks	Cognitive function (Test of Gross Motor Development–Second Edition)	Cognitive function differed significantly be-tween IG and CG	<0.01
2024	Kim et al., [56]	N = 31 (CG = 15 and IG = 16) M = 26/F = 5 13–15 years	CG: Regular physical education class but no PA intervention	IG: Frequency: 3 times/week Type: TT exercises Session length: 90 min Intervention duration: 8 weeks	Cognitive function (Korean Developmental Test of Visual Perception for Adolescents), Balance function (Bruininks–Oseretsky Test of Motor Proficiency—Second Edition)	Cognitive function and balance function differed significantly between IG and CG	<0.001
2010	Zhang and Gao, [54]	N = 308 (CG = 156 and IG = 152) M = NA/F = NA 7–9 years	CG: Regular physical education class but no TT intervention	IG: Frequency: 2–3 times/week Type: TT exercises Session length: 40 min Intervention duration: 12 weeks	Balance function (Movement ABC test)	Balance function differed significantly between IG and CG	<0.05

Note. PA: physical activity, TT: table tennis, CG: control group, IG: intervention group, M: male, F: female, NA: not available, TIME-SLC: Single-leg stance with eyes closed, SPPB: Short Physical Performance Battery, BOT-2: Bruininks–Oseretsky Test of Motor Proficiency, Second Edition (Body Coordination Composite—Balance), ABC: Movement Assessment Battery for Children (Movement ABC test).

**Table 2:** Results of the sensitivity analysis for balance function.

Study	SMD	95% CI	I^2^	*p*
Kim et al., 2024 [56]	0.70	[0.51, 0.88]	54%	<0.001
Liu, 2020 [48]	0.78	[0.56, 0.99]	66%	<0.001
Ma et al., 2021 [49]	0.73	[0.53, 0.94]	62%	<0.001
Naderi et al., 2021 [57]	0.73	[0.53, 0.93]	61%	<0.001
Pan et al., 2016 [58]	0.81	[0.60, 1.02]	65%	<0.001
Pan et al., 2017 [30]	0.78	[0.56, 0.99]	66%	<0.001
Tian and Du, 2016 [50] IG1	0.86	[0.59, 1.13]	66%	<0.001
Tian and Du, 2016 [50] IG2	0.88	[0.61, 1.14]	66%	<0.001
Yun et al., 2017a [52]	0.75	[0.55, 0.96]	64%	<0.001
Yun et al., 2017b [53] IG1	0.76	[0.55, 0.96]	65%	<0.001
Yun et al., 2017b [53] IG2	0.76	[0.56, 0.97]	66%	<0.001
Zhang and Gao, 2010 [54]	0.85	[0.64, 1.07]	56%	<0.001

Note. SMD: standardized mean difference, CI: confidence interval. IG1: intervention group 1, IG2: intervention group 2.

**Table 3:** Results of the sensitivity analysis for cognitive function.

Study	SMD	95% CI	I^2^	*p*
Chang et al., 2022 [55]	2.12	[1.30, 2.94]	97%	<0.001
Hu and Wang, 2014 [47]	2.22	[1.54, 2.90]	94%	<0.001
Kim et al., 2024 [56]	2.06	[1.23, 2.89]	97%	<0.001
Ma et al., 2021 [49]	1.85	[1.03, 2.67]	97%	<0.001
Pan et al., 2015 [59]	2.20	[1.41, 2.99]	96%	<0.001
Pan et al., 2016 [58]	2.00	[1.16, 2.83]	97%	<0.001
Tian and Du, 2016 [50] IG1	1.87	[0.97, 2.76]	95%	<0.001
Tian and Du, 2016 [50] IG2	1.93	[0.87, 2.98]	97%	=0.003
Yang et al., 2025 [51]	2.16	[1.34, 2.97]	96%	<0.001
Yun et al., 2017b [53]	2.15	[1.34, 2.97]	97%	<0.001

Note. SMD: standardized mean difference, CI: confidence interval. IG1: intervention group 1, IG2: intervention group 2.

**Table 4:** Results of subgroup analyses based on intervention population, health status, intervention duration, and session length.

Subgroup		Categories	Number of Studies	SMD	95% CI	I^2^	*p*
Balance function	Intervention population	Children	5	0.74	[0.23, 1.26]	72%	=0.005
		Older adults	7	0.80	[0.59, 1.01]	53%	<0.001
	Health status	Healthy individuals	9	0.72	[0.53, 0.92]	60%	<0.001
		Non-healthy individuals	3	0.95	[−0.04, 1.94]	78%	=0.06
	Intervention duration	≤12 weeks	8	0.88	[0.47, 1.30]	67%	<0.001
		>12 weeks	4	0.73	[0.51, 0.95]	65%	<0.001
	Session length	≤60 min	5	0.84	[0.37, 1.31]	60%	=0.005
		>60 min	4	1.06	[0.32, 1.80]	71%	=0.005
Cognitive function	Intervention population	Children	4	1.64	[0.87, 2.41]	70%	<0.001
		Older adults	6	2.30	[1.31, 3.29]	97%	<0.001
	Health status	Healthy individuals	5	2.65	[1.89, 3.41]	94%	<0.001
		Non-healthy individuals	5	1.42	[0.74, 2.10]	77%	<0.001
	Intervention duration	≤12 weeks	7	1.32	[0.84, 1.79]	66%	<0.001
		>12 weeks	3	3.46	[3.02, 3.91]	83%	<0.001
	Session length	≤60 min	4	0.96	[0.66, 1.26]	0%	<0.001
		>60 min	4	1.74	[0.62, 2.85]	80%	=0.002

Note. SMD: standardized mean difference, CI: confidence interval.

## Data Availability

No new data were created or analyzed in this study. Data sharing is not applicable to this article.
